# Coexistence of cutaneous endometriosis and ovarian endometrioma: a case report

**DOI:** 10.1186/s13256-022-03483-8

**Published:** 2022-07-03

**Authors:** Fatemeh Mohaghegh, Parvaneh Hatami, Parvin Rajabi, Zeinab Aryanian

**Affiliations:** 1 Department of Dermatology, Skin Diseases and Leishmaniasis Research Center, Isfahan University of Medical Sciences, Isfahan, Iran; 2 Autoimmune Bullous Diseases Research Center, Razi Hospital, Tehran University of Medical Sciences, Tehran, Iran; 3 Department of Pathology, Isfahan University of Medical Sciences, Isfahan, Iran; 4 Department of Dermatology, Babol University of Medical Sciences, Babol, Iran

**Keywords:** Cutaneous endometriosis, Umbilical endometriosis, Endometrioma, Ovarian cyst

## Abstract

**Background:**

Umbilical endometriosis is a rare entity accounting for 0.5–4% of cases with endometriosis.

**Case presentation:**

Here we report a rare case of umbilical endometriosis with concurrent ovarian endometriomas in a 37-year old primiparous Iranian woman.

**Conclusion:**

This interesting coexistence reflects the importance of thorough gynecological assessment in patients with cutaneous endometriosis to enable appropriate management.

## Introduction

Endometriosis is a common gynecologic condition characterized by the presence of endometrial tissue in anatomical sites other than the uterus and can lead to chronic pelvic pain or even infertility in women. The most common sites of involvement are ovaries followed by the Douglas pouch and pelvic ligaments, respectively [1]. It can be rarely found in other organs such as skin. Umbilical endometriosis is an unusual condition accounting for 0.5–1% of extra pelvic endometriosis [2]. Here we report a case of umbilical endometriosis with concurrent ovarian endometriomas.

## Case presentation

A 37-year old primiparous Iranian woman presented to our dermatology clinic with a complaint of an asymptomatic lesion in the umbilicus. The lesion appeared 1.5 years ago and had slightly increased in size. She denied previous piercing or trauma. However, she had had a laparoscopic cholecystectomy with concurrent ovarian dermoid cystectomy about 10 years ago. She had regular menstrual cycles with normal flow.

**Fig. 1:**
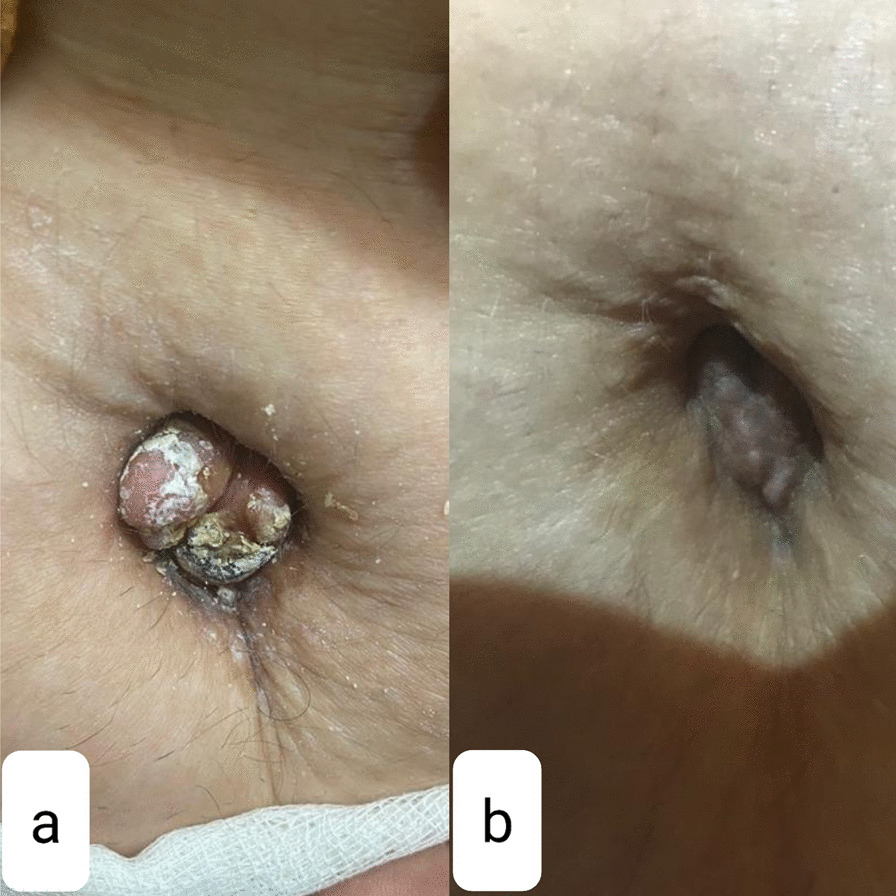
A bilubolated umbilical mass, before (**a**) and after (**b**) treatment

**Fig. 2:**
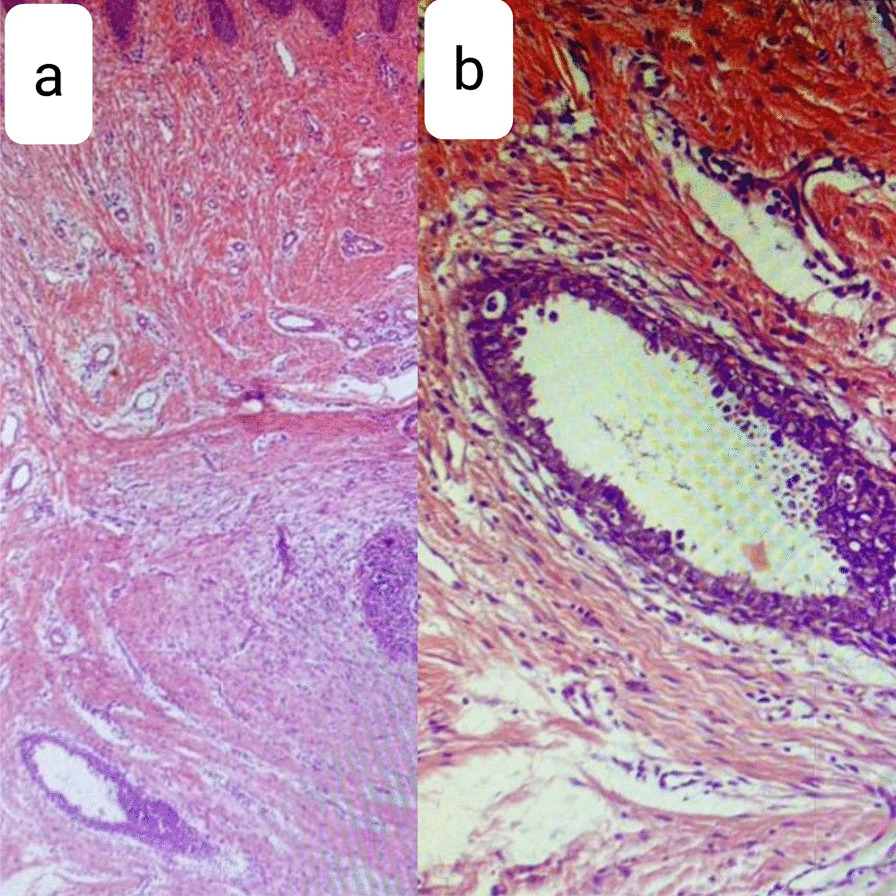
Endometrial glandular structures surrounded by scant hypercellular stroma. **a** H&E ×40, **b** H&E ×100

**Fig. 3:**
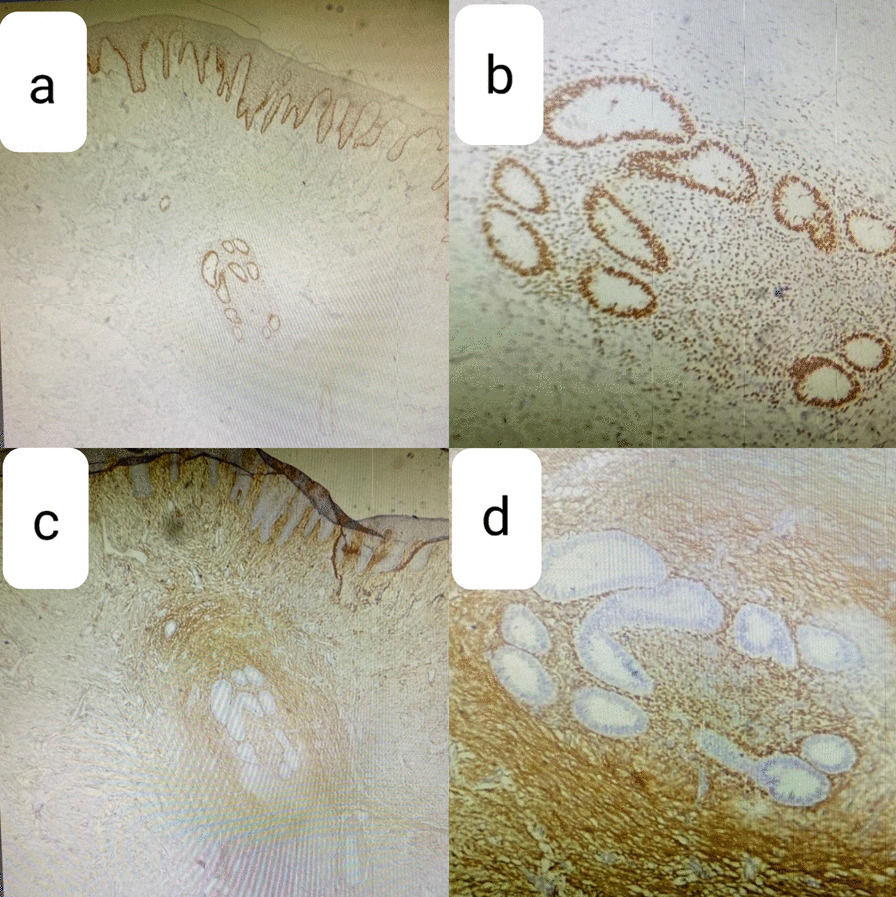
Immunohistochemistry staining for estrogen receptor (×40, ×100) (**a**, **b**) and CD10 (×40, ×100) (**c**, **d**)

Physical examination revealed a well-circumscribed, firm, bilobulated 20 × 22 mm^2^, nontender nodule on her umbilicus (Fig. 1a). An incisional biopsy was performed with differential diagnosis of adnexal tumor, umbilical granuloma, and metastasis, but surprisingly, histopathologic assessment showed the presence of a few dilated glands with stratified columnar epithelium in secretory phase (Fig. 2). The glands were surrounded by hypercellular stroma. Immunohistochemistry (IHC) staining showed positivity for estrogen receptor in the nuclei of epithelial cells lining glandular structure endometrial-like cells, CD10 diffuse and intense positivity in the stroma, and Ki67 positivity below 1% of epithelial cell nuclei (Fig. 3). These findings confirmed the diagnosis of cutaneous endometriosis. No signs of atypia or malignancy were observed. After consultation with gynecology service, abdominopelvic sonography was conducted, which showed an increased density at the umbilicus and also multiple ovarian cysts with appearance compatible with endometrioma (two 15 × 10 mm^2^ cysts in right ovary and a 35 × 48 mm^2^ cyst in left one).

After establishing the diagnosis, total umbilectomy was suggested. However, due to the patient’s phobia regarding that, just the nodule was excised as completely as possible by dermatologist, and oral progestin (Dienogest) was initiated. The remnants of the umbilical lesion had dramatically improved after 1 month (Fig. 1b). Follow-up is in progress.

## Discussion

Endometriosis is a benign disease with incidence of 6–10% in women of childbearing age [1], usually being seen in the pelvic area. Cutaneous endometriosis can appear in less than 5% of cases [2], most of which have a history of former surgery [3]. Umbilical endometriosis appeared in 0.5–4% of affected cases, usually presenting with painful umbilical mass with periodic discharge or bleeding. An interesting feature of this case is that the lesion was totally asymptomatic. Another prominent feature is the presence of bilateral ovarian endometrioma, which reveals the importance of gynecological assessment in patients with cutaneous endometriosis to identify any pelvic involvement, which is reported to occur in 15% of these patients [4].

Regarding treatment of cutaneous endometriosis, complete excision is considered as the treatment of choice with or without hormonal therapy for ameliorating the symptoms [5–7].

Ovaries are the most common sites of endometriosis [8], and ovarian endometrioma accounts for 35% of all benign ovarian cysts [9]. Interestingly, ovarian endometriomas are more frequent in the left versus right ovary, possibly due to anatomical asymmetry and compression leading to venous congestion and hypoxia in the left side, affecting release of cytokines and sex hormones [10, 11]. In our case, endometriomal cyst in left ovary was considerably larger than on the other side, which might be in favor of the above-mentioned theory.

In addition to pain, discomfort, and fertility issues, an increased risk of infection, rupture, or transformation into ovarian cancer [12] obligates surgical intervention in larger ovarian endometriomas [9]. Nonsteroidal antiinflammatory drugs, gonadotropin-releasing hormone (GnRH) agonists, and progestins are also considered as mainstream therapeutic options [9].

## Conclusion

Umbilical endometriosis is a rare entity that might occur with concurrent pelvic endometriosis. Hence, thorough gynecological assessment is necessary in such patients to enable proper management.

## Data Availability

The data that support the findings of this study are available from the corresponding author upon reasonable request.
